# Association Between Serum Osmolality and Acute Kidney Injury in Critically Ill Patients: A Retrospective Cohort Study

**DOI:** 10.3389/fmed.2021.745803

**Published:** 2021-10-15

**Authors:** Jie Yang, Yisong Cheng, Ruoran Wang, Bo Wang

**Affiliations:** Department of Critical Care Medicine, West China Hospital, Sichuan University, Chengdu, China

**Keywords:** critical care, AKI (acute kidney injury), serum osmolality, independent risk factor, outcome

## Abstract

**Purposes:** Acute kidney injury (AKI) is a common complication in critically ill patients and is usually associated with poor outcomes. Serum osmolality has been validated in predicting critically ill patient mortality. However, data about the association between serum osmolality and AKI is still lacking in ICU. Therefore, the purpose of the present study was to investigate the association between early serum osmolality and the development of AKI in critically ill patients.

**Methods:** The present study was a retrospective cohort analysis based on the medical information mart for intensive care III (MIMIC-III) database. 20,160 patients were involved in this study and divided into six subgroups according to causes for ICU admission. The primary outcome was the incidence of AKI after ICU admission. The association between early serum osmolality and AKI was explored using univariate and multivariate logistic regression analyses.

**Results:** The normal range of serum osmolality was 285–300 mmol/L. High serum osmolality was defined as serum osmolality >300 mmol/L and low serum osmolality was defined as serum osmolality <285 mmol/L. Multivariate logistic regression indicated that high serum osmolality was independently associated with increased development of AKI with OR = 1.198 (95% CL = 1.199–1.479, *P* < 0.001) and low serum osmolality was also independently associated with increased development of AKI with OR = 1.332 (95% CL = 1.199–1.479, *P* < 0.001), compared with normal serum osmolality, respectively.

**Conclusions:** In critically ill patients, early high serum osmolality and low serum osmolality were both independently associated with an increased risk of development of AKI.

## Introduction

Acute kidney injury (AKI) is a common clinical syndrome characterized by a quick decrease in renal function within a short time, with an obvious accumulation of creatinine and urea or a decrease in urinary output (1, 2). The primary potential pathogenesis of AKI may be renal cell injury due to unstable hemodynamics, systemic inflammation, or sepsis (3). AKI is frequent in hospitalized patients and especially common in critically care ill patients (4–6). Previous studies reported that the development of AKI was commonly associated with patient poor outcomes including prolonged length of hospital stay and increased mortality (7, 8). Therefore, the number of investigations and researches on AKI in critically ill patients was continuously increasing and relevant results were discovered. Such as trauma, increased blood lactate, shock, old age, red blood cell distribution width (RDW), and increased procalcitonin were investigated to be independent risk factors for the development of AKI (9–12).

Serum osmolality is the serum concentration of ions and particles dissolved in body fluid to reflect the body fluid balance and renal function, and is strongly affected by the concentration of Na^+^, K^+^, glucose, and urea (13–15). Therefore, many studies about serum osmolality affecting patient outcomes were conducted. Shen and his colleagues reported that the association between serum osmolality and mortality presented a U-shaped curve in critically ill patients (high osmolality and low osmolality were both associated with increased mortality) (16). Masanari and his (her) team found that elevated serum osmolality was an independent risk factor for developing chronic kidney disease (17). Elevated serum osmolality on intensive care unit (ICU) admission was also associated with an increased risk of critically ill patient mortality (18). In summary, serum osmolality is a useful and valuable indicator to predict or reflect patient outcomes in hospitalized patients and critically ill patients.

Even though several studies about serum osmolality were conducted and proved that abnormal serum osmolality was an independent risk factor for poor outcomes, data on the relationship between early serum osmolality on ICU admission and the development of AKI in critically ill patients has not been explored and validated. Therefore, the present study was designed and conducted to investigate the association between early serum osmolality on ICU admission and AKI in critically ill patients, and subgroup analyses were performed according to causes for ICU admission.

## Materials and Methods

### Database Source

Medical information mart for intensive care III (MIMIC-III, version 1.4) database is a single-center and big database consisting of related information about 61,532 ICU stays (53,432 stays for adult patients and 8,100 for neonatal patients) at a large tertiary hospital from 2001 to 2012. The MIMIC-III database includes patient demographics, clinical measurements, clinical laboratory tests, interventions, pharmacotherapy, medical data, survival data, and more (19). The researchers who completed and passed the required training course could acquire access to this database. The consent for original data acquisition was obtained and the institutional review boards of the Massachusetts Institute of Technology approved the establishment of the database. Thus, patient informed consent and ethics approval were inapplicable for the present study. Data collected and presented in this study were extracted by the author Yang and Cheng who completed and passed the required training course.

### Study Population and Data Collection

We conducted a retrospective cohort study based on the MIMIC-III database. Patients older than 18 years old were involved in the present study. Patients were excluded if they had been diagnosed with AKI on ICU admission and patients without sufficient data to calculate serum osmolarity were also excluded.

Patient medical information was extracted and collected using PostgreSQL tools version 13.0. The data were collected including demographics and characteristics, signs and symptoms, comorbidities, causes for admission, laboratory findings, treatment, and outcomes. Except for patient outcomes, the rest of the variables were collected on patient ICU admission. The primary outcome in the present study was the incidence of AKI after enrolled patients on ICU admission. AKI was diagnosed according to “KDIGO Clinical Practice Guidelines”: increase in serum creatinine by ≥0.3 mg/dl (or ≥ 26.5 μmol/l) in 48 h, increase in serum creatinine to 1.5 times over baseline in 7 days, and patient urine output ≤ 0.5 ml/kg/h for 6 h. Other secondary outcomes included length of ICU stay, length of hospital stay, ICU mortality, hospital mortality, and use of vasopressor (20).

Serum osmolality was calculated by the following equation: (2 × Na^+^ + K^+^) + (glucose/18) + (urea/2.8) (15). The values of Na^+^, K^+^, glucose, and urea measured at the same time were involved in serum osmolality calculation. In the MIMIC III database, many results of serum osmolality were calculated by the above equation in each critically ill patient within the corresponding ICU record, and the first serum osmolality in this ICU record was selected to reflex the early serum osmolality on each patient ICU admission. The normal range of serum osmolality in the present investigation was defined as 285–300 mmol/L according to a previous study reported in “The New England Journal of Medicine” (21). Therefore, high serum osmolality was defined as serum osmolality >300 mmol/L, low serum osmolality was defined as serum osmolality <285 mmol/L, and a normal range of 285–300 mmol/L was used as the reference group. Subgroup analyses were also conducted based on causes for ICU admission, including sepsis, respiratory diseases, cardiac diseases, cerebral diseases, hepatic diseases, and cancer.

### Statistical Analyses

Continuous variables were expressed as mean ± standard deviation (SD) or median and interquartile range (IQR) according to their distribution. Independent *t*-test was used for normal distribution or Mann–Whitney *U* test was used for skewed distribution to compare differences. Categorical variables were expressed as numbers and percentages, and compared by Chi-square or Fisher's exact probability test as appropriate. Univariate and multivariate logistic regression analyses (variables presenting *P* < 0.05 in univariate analysis were included in multivariate logistic regression) were used to identify the associations between high or low serum osmolality and the development of AKI. The Lowess smoothing was also used to explore the curve relationship between serum osmolality and the incidence of AKI, and crude relationships between serum osmolality and AKI in six subgroups divided by causes for ICU admission. *P* < 0.05 was considered statistically significant in comparing differences between two groups or logistic regression models. All statistical analyses in the present study were performed using SPSS version 22.0, STATA version 16.0, and R software version 4.0.4.

## Results

**Figure 1:**
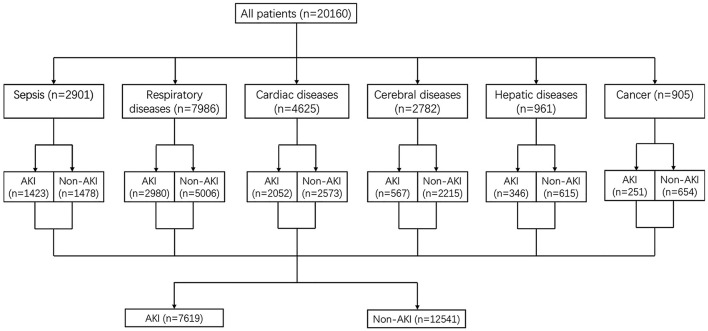
Study population.

There were 20,160 critically ill patients involved in this study. The involved patients were divided into six subgroups based on causes for ICU admission, such as sepsis, respiratory diseases, cardiac diseases, cerebral diseases, hepatic diseases, and cancer (shown in Figure 1). 7,619 patients developed AKI after ICU admission and 12,541 patients did not develop AKI in this ICU treatment stage. All patients' median age was 67.3 years old and the median age of patients diagnosed with AKI was significantly older than patients without AKI (68.8 vs. 54.0, *P* < 0.001). The median SOFA score in the AKI group was significantly higher than the non-AKI group (5.0 vs. 3.0, *P* < 0.001). Proportions of high serum osmolality and low serum osmolality in the AKI group were both higher than the non-AKI group (39.0 vs. 30.2% and 10.8 vs. 9.3%, *P* < 0.001). Other secondary outcomes including length of ICU stay, length of hospital stay, ICU mortality, and hospital mortality were all longer or higher in patients with AKI (*P* < 0.001). Other comparisons of demographics and clinical characteristics between the non-AKI group and the AKI group were shown in Table 1.

**Table 1:** Comparisons of demographics between non-AKI and AKI.

**Variables**	**Total (*n* = 20,160)**	**Non-AKI (*n* = 12,541)**	**AKI (*n* = 7,619)**	***P* value**
**Demographics and characteristics**
Age, year, median (IQR)	67.3 (55.0, 78.9)	54.0 (66.3, 78.6)	68.8 (56.8, 79.3)	<0.001
Male, no. (%)	10,678 (53.0)	6,583 (52.5)	4,095 (53.7)	0.083
Weight, kg, median (IQR)	77.6 (65.0, 91.3)	77.4 (65.0, 90.7)	77.9 (65.2, 92.6)	0.146
Height, cm, median (IQR)	167.7 (163.9, 172.9)	167.7 (164.2, 172.7)	167.6 (162.9, 173.6)	0.507
BMI, median (IQR)	27.3 (23.7, 31.2)	27.2 (23.7, 31.0)	27.4 (23.7, 31.6)	0.069
SOFA, median (IQR)	4.0 (2.0, 6.0)	3.0 (1.0, 5.0)	5.0 (3.0, 8.0)	<0.001
**Signs and symptoms**
Respiratory rate, median (IQR)	18.0 (14.0, 22.0)	18.0 (14.0, 22.0)	18.0 (14.0, 23.0)	0.014
Systolic pressure, mmHg, median (IQR)	123.0 (107.0, 141.0)	124.0 (108.0, 143.0)	118.0 (102.0, 137.0)	<0.001
Diastolic pressure, mmHg, median (IQR)	63.0 (54.0, 74.0)	64.0 (54.0, 76.0)	61.0 (51.0, 72.0)	<0.001
MAP, mmHg, median (IQR)	83.0 (73.0, 95.0)	85.0 (75.0, 97.0)	81.0 (71.0, 92.0)	<0.001
Heart rate, median (IQR)	89.0 (84.0, 93.0)	89.0 (84.0, 94.0)	89.0 (85.0, 93.0)	<0.001
Temperature, °C, median (IQR)	36.6 (36.1, 37.2)	36.6 (36.1, 37.2)	36.6 (35.9, 37.2)	<0.001
**Comorbidities**
Hypertension, no. (%)	9,683 (48.0)	6,177 (49.3)	3,506 (46.0)	<0.001
Diabetes, no. (%)	1,969 (9.8)	1,134 (9.0)	835 (11.0)	<0.001
COPD, no. (%)	657 (3.3)	408 (3.3)	249 (3.3)	0.954
**Causes for admission**
Sepsis, no. (%)	2,901 (14.4)	1,478 (11.8)	1,423 (18.7)	<0.001
Respiratory diseases, no. (%)	7,986 (39.6)	5,006 (39.9)	2,980 (39.1)	0.258
Cardiac diseases, no. (%)	4,625 (22.9)	2,573 (20.5)	2,052 (26.9)	<0.001
Cerebral diseases, no. (%)	2,782 (13.8)	2,215 (17.7)	567 (7.4)	<0.001
Hepatic diseases, no. (%)	961 (4.8)	615 (4.5)	346 (4.5)	0.241
Cancer, no. (%)	905 (4.5)	654 (5.2)	251 (3.3)	<0.001
**Laboratory findings**
WBC count, × 10^9^/L, median (IQR)	11.0 (7.9, 14.9)	10.7 (7.8, 14.5)	11.5 (8.0, 15.8)	<0.001
Platelet count, × 10^9^/L, median (IQR)	205.0 (145.0, 274.0)	211.0 (156.0, 278.0)	190.0 (129.0, 265.0)	<0.001
Hemoglobin, g/dL, median (IQR)	10.9 (9.5, 12.4)	11.2 (9.8, 12.6)	10.5 (9.2, 12.0)	<0.001
Creatinine, mg/dL, median (IQR)	0.9 (0.7, 1.2)	0.9 (0.7, 1.1)	1.0 (0.7, 1.6)	<0.001
Blood glucose, mg/dL, median (IQR)	131.0 (107.0, 166.0)	128.0 (106.0, 161.0)	135.0 (109.0, 173.0)	<0.001
Sodium, mmol/L, median (IQR)	138.0 (136.0, 141.0)	139.0 (136.0, 141.0)	138.0 (135.0, 141.0)	<0.001
Potassium, mmol/L, median (IQR)	4.0 (3.7, 4.5)	4.0 (3.7, 4.4)	4.1 (3.7, 4.7)	<0.001
Urea, mg/dL, median (IQR)	18.0 (13.0, 28.0)	17.0 (12.0, 24.0)	22.0 (15.0, 36.0)	<0.001
Serum osmolarity, mmol/L, median (IQR)	296.0 (290.0, 303.0)	296.0 (290.0, 302.0)	297.0 (290.0, 306.0)	<0.001
Serum osmolarity, no. (%)				<0.001
<285 mmol/L	1,989 (9.9)	1,163 (9.3)	826 (10.8)	
285–300 mmol/L	11,418 (56.6)	7,595 (60.6)	3,823 (50.2)	
>300 mmol/L	6,753 (33.5)	3,783 (30.2)	2,970 (39.0)	
Urine within first 24 h, mL, median (IQR)	1,700.0 (1051.5, 2544.5)	1,815.0 (1175.0, 2638.0)	1505.0 (850.0, 2370.0)	<0.001
**Treatment**
Albumin, no. (%)	1,304 (6.5)	439 (3.5)	865 (11.4)	<0.001
Norepinephrine, no. (%)	1,312 (6.5)	516 (4.1)	796 (10.4)	<0.001
**Outcomes**
ICU LOS, days, median (IQR)	2.6 (1.4, 5.3)	2.1 (1.2, 4.0)	3.7 (1.9, 7.9)	<0.001
Hospital LOS, days, median (IQR)	7.0 (4.1, 12.2)	5.9 (3.7, 10.0)	9.0 (5.8, 15.8)	<0.001
ICU mortality, no. (%)	2,161 (10.7)	889 (7.1)	1,272 (16.7)	<0.001
Hospital mortality, no. (%)	2,882 (14.3)	1,217 (9.7)	1,665 (21.9)	<0.001

*AKI, acute kidney injury; IQR, interquartile range; BMI, body mass index; MAP, mean arterial pressure; COPD, chronic obstructive pulmonary disease; SOFA, sequential organ failure assessment; WBC, white blood cell; LOS, length of stay*.

**Figure 2:**
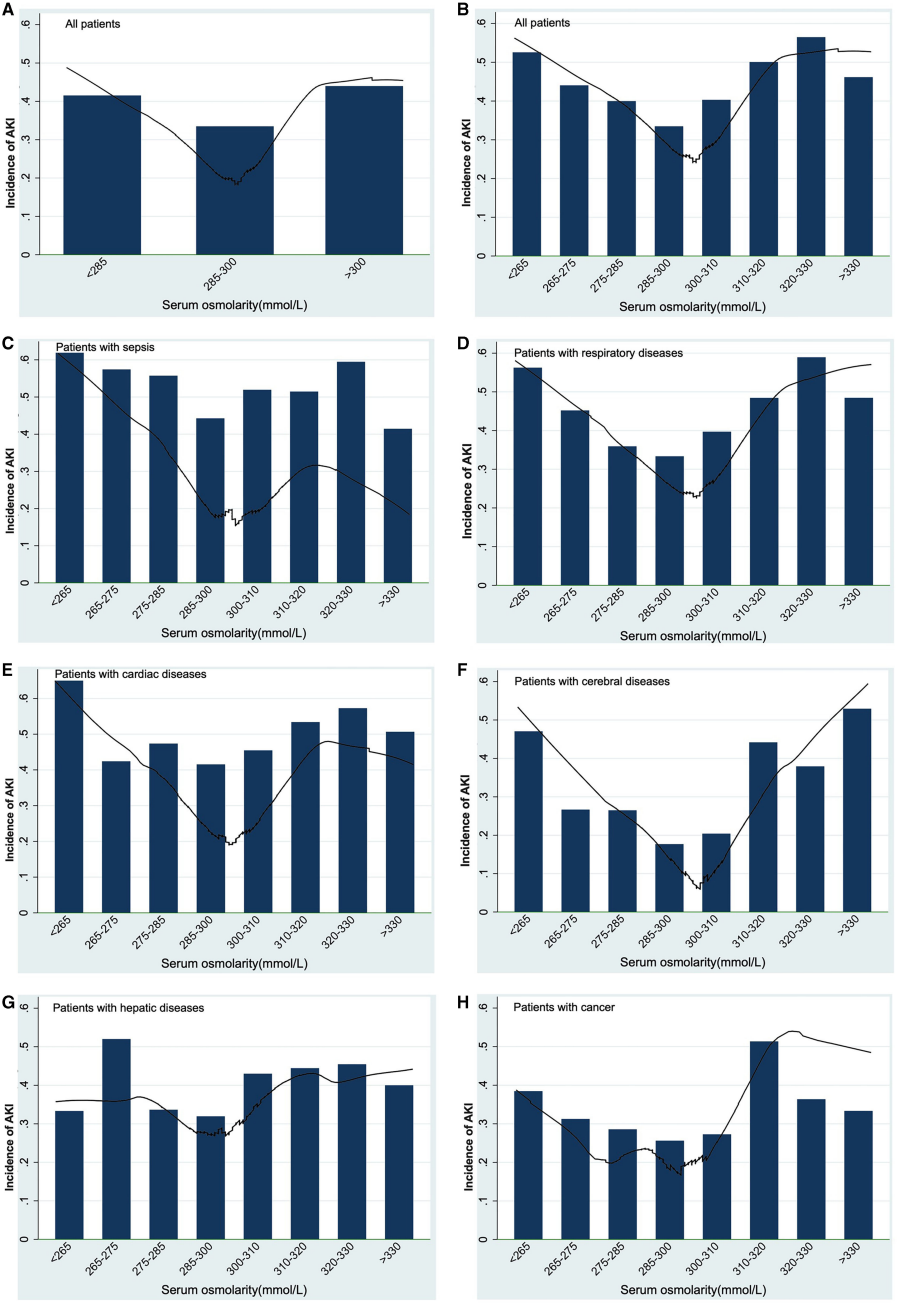
Association between serum osmolality and the development of AKI using Lowess smoothing: **(A,B)**, all patients; **(C)** patients with sepsis; **(D)** patients with respiratory diseases; **(E)** patients with cardiac diseases; **(F)** patients with cerebral diseases; **(G)** patients with hepatic diseases; **(H)** patients with cancer. Non-liner relationships (U-shaped curves) were exhibited in the present figure.

**Figure 3:**
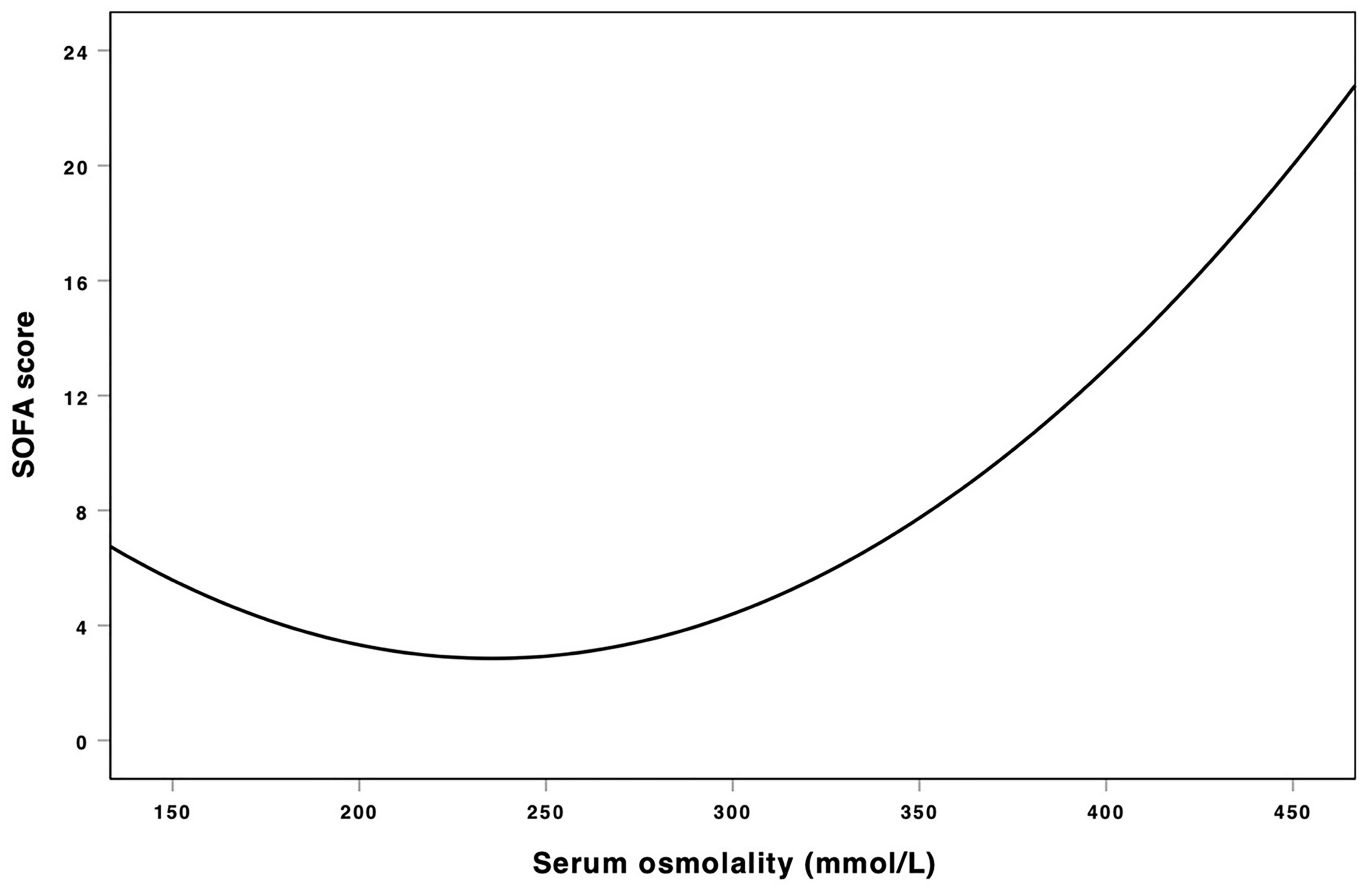
Association between serum osmolality and SOFA score. Non-liner relationships (U-shaped curves) were exhibited in the present figure.

**Figure 4:**
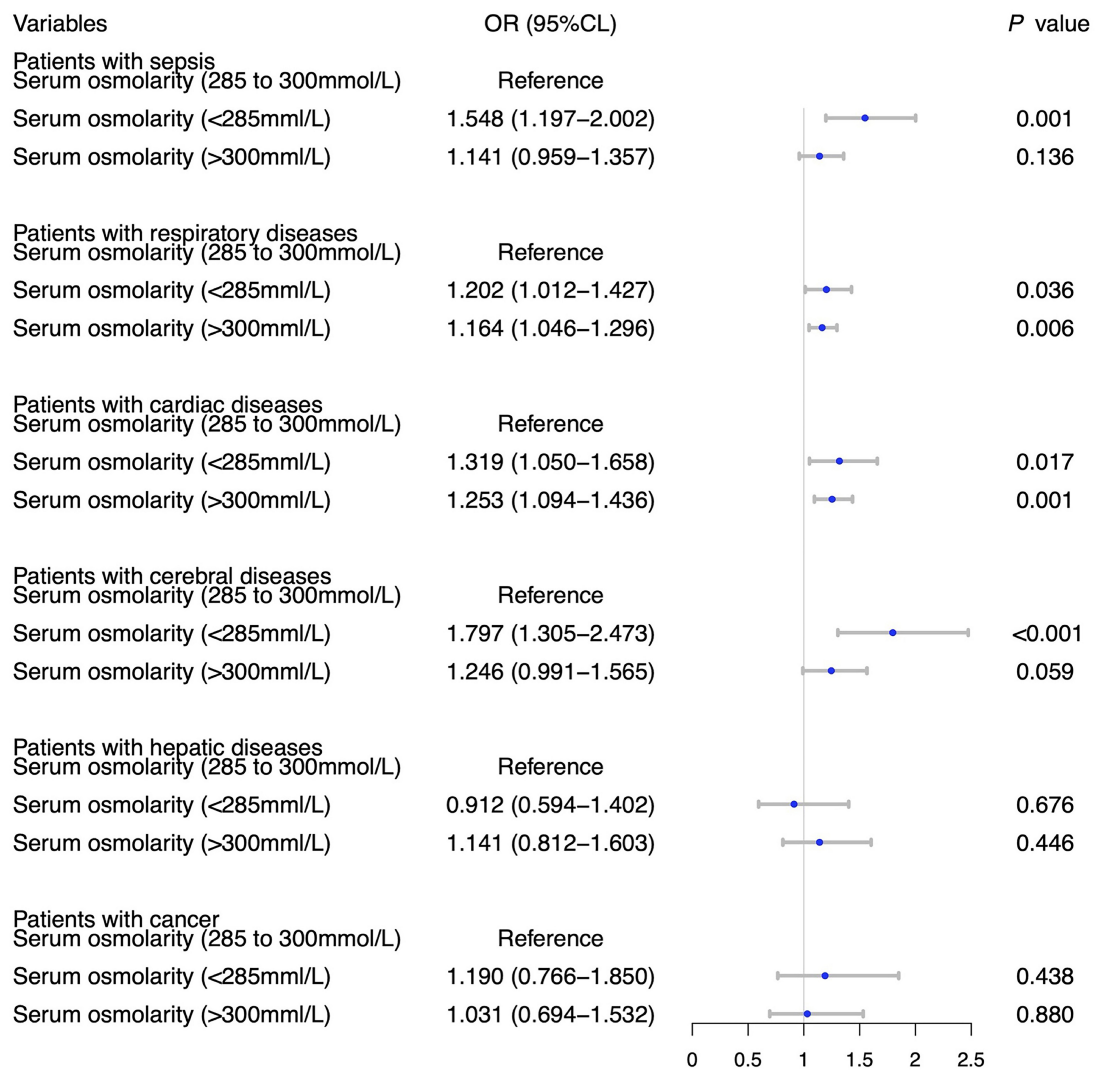
Subgroup analyses on association between serum osmolality and the development of AKI based on causes for ICU admission with adjustment for age, BMI, MAP, temperature, hypertension, diabetes, SOFA, WBC count, platelet count, hemoglobin, use of albumin, and use of norepinephrine.

Table 2 showed the results of univariate and multivariate logistic analyses. High serum osmolality (>300 mmol/L) was an independent risk factor for the development of AKI (OR = 1.332, 95% CL = 1.199–1.479, *P* < 0.001) and low serum osmolality (<285 mmol/L) was also an independent risk factor for the development of AKI (OR = 1.198, 95% CL = 1.119–1.283, *P* < 0.001), compared with normal serum osmolality (285–300 mmol/L). Figure 2 using the Lowess smoothing demonstrated the non-line relationship between serum osmolality and the incidence of AKI. A U-curve relationship between serum osmolality and the incidence of AKI was found in Figures 2A,B. High serum osmolality or low serum osmolality were both associated with increased risk for AKI. This U-curve relationship (Figures 2A,B) was consistent with multivariate logistic analysis. We also explored the crude relationship between serum osmolality and the incidence of AKI in six subgroups using Lowess smoothing. The approximately U-curve relationships between serum osmolality and the incidence of AKI were also found in six subgroups shown in Figures 2C–H. The U-curve relationship between serum osmolality and SOFA score was presented in Figure 3. We further conducted subgroup analyses based on causes for ICU admission to determine whether serum osmolality also affected the development of AKI in different diseases. Figure 4 showed that abnormal serum osmolality was independently associated with the development of AKI in sepsis, respiratory diseases, cardiac diseases, and cerebral diseases after adjustment for related covariates (*P* < 0.05).

**Table 2:** Univariate and multivariate logistic regression analyses for AKI.

	**Univariate logistic analysis**		**Multivariate logistic analysis**	
**Variables**	**OR (95% CL)**	***P* value**	**OR (95% CL)**	***P* value**
Age, year	1.008 (1.006–1.009)	<0.001	1.004 (1.002–1.006)	<0.001
BMI	1.006 (1.002–1.010)	0.008	1.007 (1.002–1.011)	0.009
MAP, mmHg	0.985 (0.984, 0.987)	<0.001	0.999 (0.997–1.001)	0.367
Temperature, °C	0.917 (0.893–0.942)	<0.001	0.970 (0.942–0.998)	0.038
Hypertension	0.878 (0.830–0.930)	<0.001	0.919 (0.861–0.980)	0.010
Diabetes	1.238 (1.127–1.361)	<0.001	1.192 (1.076–1.321)	0.001
Sepsis	1.719 (1.588–1.861)	<0.001	1.005 (0.914–1.106)	0.915
Cardiac diseases	1.428 (1.336–1.526)	<0.001	1.514 (1.405–1.632)	<0.001
SOFA	1.245 (1.233–1.258)	<0.001	1.212 (1.198–1.227)	<0.001
WBC count, × 10^9^/L	1.021 (1.016–1.025)	<0.001	1.008 (1.003–1.014)	0.001
Platelet count, × 10^9^/L	0.999 (0.998–0.999)	<0.001	1.000 (1.000–1.001)	0.031
Hemoglobin, g/dL	0.875 (0.863–0.888)	<0.001	0.932 (0.908–0.937)	<0.001
Albumin	3.531 (3.136–3.975)	<0.001	2.213 (1.946–2.516)	<0.001
Norepinephrine	2.719 (2.424–3.049)	<0.001	1.258 (1.103–1.433)	<0.001
**Serum osmolarity, mmol/L**
**Serum osmolarity (285–300)**	**Reference**		**Reference**	
Serum osmolarity (<285)	1.411 (1.280–1.555)	<0.001	1.332 (1.199–1.479)	<0.001
Serum osmolarity (>300)	1.560 (1.466–1.659)	<0.001	1.198 (1.119–1.283)	<0.001

*AKI, acute kidney injury; BMI, body mass index; MAP, mean arterial pressure; SOFA, sequential organ failure assessment; WBC, white blood cell*.

The crude associations between serum osmolality and secondary outcomes were explored without adjustment for related covariates (shown in Table 3). ICU length of stay and hospital length of stay in the groups of high or low serum osmolality were both longer than the group of normal serum osmolality (*P*_1_ <0.001 and *P*_2_ <0.001). ICU mortality and hospital mortality were both higher in the groups of high and low serum osmolality (*P*_1_ <0.001 and *P*_2_ <0.001). The proportion of use of vasopressor was also higher in the group of high serum osmolality (*P*_2_ <0.001).

**Table 3:** Unadjusted other outcomes by serum osmolarity categories.

	**Serum osmolarity, mmol/L**				
**Variables**	** <285**	**285–300**	**>300**	***P*_**1**_ value**	***P*_**2**_ value**
	**(*n* = 1,989)**	**(*n* = 11,418)**	**(*n* = 6,753)**		
ICU LOS, days, median (IQR)	2.6 (1.4–5.1)	2.3 (1.3–4.7)	3.0 (1.6–6.5)	<0.001	<0.001
Hospital LOS, days, median (IQR)	7.1 (4.4–13.3)	6.6 (4.1–11.1)	7.7 (4.2–13.9)	<0.001	<0.001
ICU mortality, no. (%)	213 (10.7)	808 (7.1)	1,140 (16.9)	<0.001	<0.001
Hospital mortality, no. (%)	292 (14.7)	1,124 (9.8)	1,466 (21.7)	<0.001	<0.001
Vasopressor, no. (%)	119 (6.0)	586 (5.1)	607 (9.0)	0.117	<0.001

*P_1_ value, the p value of comparisons between the group with osmolarity <285 mmol/L and the group with osmolarity in 285–300 mmol/L*.

*P_2_ value, the p value of comparisons between the group with osmolarity >300 mmol/L and the group with osmolarity in 285–300 mmol/L*.

*LOS, length of stay; IQR, interquartile range*.

## Discussion

The incidence of AKI in critically ill patients in the present study was 37.8%, similar to the incidences reported in previous studies (22–24). We investigated the association between serum osmolality and the development of AKI in this study. The results of this study demonstrated that high serum osmolality and low serum osmolality were both independently associated with an increased risk of AKI in critically ill patients, compared with normal serum osmolality. The U-curve relationships between serum osmolality and AKI, either or SOFA score were also found. Further subgroup analyses based on causes for ICU admission showed that high or low serum osmolality were independently associated with an increased incidence of AKI in critically ill patients with sepsis, respiratory diseases, cardiac diseases, and cerebral diseases. These subgroup analyses could explain why abnormal serum osmolality was an independent risk factor for AKI in critically ill patients to some extent. The univariate analysis without adjustment for covariates showed that high osmolality or low serum osmolality were potentially associated with other outcomes. Therefore, patient serum osmolality could be considered as a potentially useful and valuable risk factor or predictor for AKI, even other patient outcomes.

In the present study, serum osmolalities >300 mmol/L or <285 mmol/L were defined as high or low serum osmolality, and abnormal serum osmolality was independently associated with an increased risk of developing AKI. Serum osmolality was a typical indicator to represent body fluid balance and renal function, and was usually calculated by the concentration of Na+, K+, glucose, and urea (25, 26). Thus, the level of these ions and particles above mentioned may reflect and affect patient renal function. Sodium imbalance was generally associated with patient poor outcomes including the development of AKI, and previous studies had proved this finding (27, 28). Relevant studies also found that early treatment for hyperkalemia could reduce potassium level and AKI, and potassium level was closely related to patient renal function (29–32). As well as sodium and potassium, the normal glucose level is important for normal physiological functions, such as reducing microvascular and macrovascular complications and infection (33). However, abnormity of glucose metabolism is frequent in critically ill patients and affects patient outcomes (34–36). Previous studies have investigated that abnormal glucose metabolism was associated with the development of AKI, and this finding may be related to increased release of inflammatory cytokines or increased inflammatory response (37–39). Therefore, appropriate control of glucose level is pivotal to maintain normal renal function and prevent AKI. Urea is generated via urea cycle enzymes mainly in the liver and subsequently eliminated in terms of urine via the kidney (40). A previous study demonstrated that the blood urea nitrogen was a useful biomarker and usually used to evaluate patient renal function (41). If renal function is injured and deficient, the capacity of excretion could be reduced and blood urea nitrogen level may elevate. Urea (blood urea nitrogen) is a potential predictor for AKI. However, with the development of research, the use of urea alone may be not a very reliable marker for AKI due to many factors affecting the excretion of urea (such as tissue breakdown, protein ingestion, and age) (41–43). Therefore, the exploration of a new comprehensive indicator for the development of AKI is important. Because imbalances of sodium, potassium, glucose, or urea were common in critically ill patients and associated with renal function, serum osmolality calculated by these particles may be a potential risk factor for developing AKI in critically ill patients. Clinicians could evaluate patient renal function preliminarily using serum osmolality on patient ICU admission and prevent developing AKI by timely appropriate treatment.

Because the causes for ICU admission in critically ill patients were complicated, we conducted subgroup analyses based on causes for ICU admission to explore the exact association between serum osmolality and AKI in different diseases. In critically ill patients with sepsis, respiratory diseases, cardiac diseases, and cerebral diseases, high osmolality or low serum osmolality were risk factors for AKI. Therefore, we should pay more attention to the abnormal changes of serum osmolality in sepsis, respiratory diseases, cardiac diseases, and cerebral diseases. The relationships between serum osmolality and other outcomes were also explored using univariate analyses. We found that high osmolality or low serum osmolality not only affected the development of AKI but also might affect patient ICU/hospital length of stay and ICU/hospital mortality, consistent with previous studies (16, 44). However, these explored results were from univariate analyses without adjustment for related covariates and lacked the power of persuasion.

Because the present study was based on the MIMIC-III database with a large sample, we could adjust for more covariates, conduct subgroup analyses, and get more information. However, limitations also existed in this study. Firstly, the present study was a retrospective study and further prospective investigation was necessary. Secondly, serum osmolality in this study was calculated by related particles and not measured directly. The association and difference in serum osmolality between calculation and direct measure were further needed exploration.

## Conclusions

In critically ill patients, early high serum osmolality and low serum osmolality were both independently associated with an increased risk of the development of AKI compared with normal serum osmolality. When patients on ICU admission and under treatment, clinicians should pay more attention to the change of serum osmolality in critically ill patients.

## Data Availability Statement

The original contributions presented in the study are included in the article/supplementary material, further inquiries can be directed to the corresponding authors.

## Ethics Statement

The consent for original data acquisition was obtained and the institutional review boards of the Massachusetts Institute of Technology approved the establishment of the database. Written informed consent for participation was not required for this study in accordance with the national legislation and the institutional requirements.

## Author Contributions

JY: study design, data extraction, data analyses, and manuscript writing. YC: data extraction and data analyses. RW: data extraction. BW: manuscript revising. All authors contributed to the article and approved the submitted version.

## Funding

This work was supported by the Project of Establishment of Early Warning and First-aid Screening System for Critically ill Patients in Hospitals Based on Artificial Intelligence (2020YFS0093) and the Project of Establishment of Treatment System Emergency and Critical Care with Cardiopulmonary Resuscitation as the Core Based on Cloud Platform (2019YFS0533).

## Conflict of Interest

The authors declare that the research was conducted in the absence of any commercial or financial relationships that could be construed as a potential conflict of interest.

## Publisher's Note

All claims expressed in this article are solely those of the authors and do not necessarily represent those of their affiliated organizations, or those of the publisher, the editors and the reviewers. Any product that may be evaluated in this article, or claim that may be made by its manufacturer, is not guaranteed or endorsed by the publisher.

## Abbreviations

AKI: acute kidney injury
MIMIC-III: medical information mart for intensive care III
RDW: red blood cell distribution width
ICU: intensive care unit
SD: standard deviation
IQR: interquartile range.
